# The Lived Experience of Thai LGBTQ_+_ Adolescents—Self-Discovery, Healing from Depression, and the Need for Support: A Phenomenological Study

**DOI:** 10.3390/ijerph22121851

**Published:** 2025-12-11

**Authors:** Wipawan Chaoum Pensuksan, Saifon Aekwarangkoon, Montha Saithanwanitkul, Christina Montsma, Earlise Ward

**Affiliations:** 1 Division of Psychiatric and Mental Health Nursing, Faculty of Nursing, Suratthani Rajabhat University, Surat Thani 84100, Thailand; wipawan.pen@sru.ac.th; 2 Division of Psychiatric and Mental Health Nursing, School of Nursing, The Excellence Center of Community Health Promotion, Walailak University, Nakhon Si Thammarat 80160, Thailand; 3 Division of Psychiatric Nursing and Mental Health, Faculty of Nursing, Thaksin University, Phathalung 93210, Thailand; montha.s@tsu.ac.th; 4 Christina Montsma Consulting and Research LLC, Ripon, WI 54971, USA; christina.montsma@gmail.com; 5 Department of Family Medicine and Community Health, School of Medicine and Public Health, University of Wisconsin-Madison, 610 N. Whitney Way, Madison, WI 53705, USA; earlise.ward@fammed.wisc.edu

**Keywords:** LGBTQ_+_ adolescents, depression, self-discovery, healing, support

## Abstract

**Highlights:**

**Public health relevance—How does this work relate to a public health issue?**

- LGBTQ_+_ adolescents face high rates of depression and mental health challenges.
- Understanding their experiences highlights opportunities for early support and intervention.

**Public health significance—Why is this work of significance to public health?**

- Provides evidence on how culture, identity development, and coping shape mental health in LGBTQ_+_ youth.
- Fills a research gap in the Thai context and suggests pathways for effective support strategies.

**Public health implications—What are the key implications or messages for practitioners, policy makers and/or researchers in public health?**

- Highlights the need for affirming schools, family support, and accessible mental health services.
- Offers guidance for inclusive policies and programs promoting positive outcomes.

**Abstract:**

LGBTQ_+_ adolescents experience disproportionately high rates of depression globally, yet little is known about how these experiences are shaped by Thailand’s unique cultural context. This study addresses this gap by examining how Thai LGBTQ_+_ adolescents understand and navigate depression, identity development, and culturally shaped support systems. A qualitative phenomenological study was conducted with a sample of 20 Thai LGBTQ_+_ adolescents from rural regions. Thematic analysis identified three interrelated themes: (1) struggles with self-discovery, acceptance, and their relationship to depression; (2) living with depression and moving forward; and (3) the need for acceptance and understanding. Findings demonstrate that cultural norms—particularly family obligations, conditional acceptance, and collectivist expectations—intensify depression and shape help-seeking, coping, and identity development. The study highlights the need for culturally responsive mental-health interventions that integrate gender-diversity awareness, family education, LGBTQ_+_-affirming school environments, and confidential support options. These findings suggest new, context-specific evidence for designing culturally responsive mental-health interventions for LGBTQ_+_ adolescents in Thailand.

## 1. Introduction

LGBTQ_+_ adolescents around the world, particularly in Southeast Asia and Thailand specifically, face disproportionately high rates of depression compared to their heterosexual peers [1]. According to the American Psychological Association (APA), LGBTQ_+_ youth are three times more likely to experience depression and anxiety than their heterosexual counterparts [2]. Recent Thai studies further reveal that depression, hopelessness, and suicidal ideation among LGBTQ_+_ adolescents are alarmingly high [2,3]. However, while these statistics reflect the scope of the problem, they neither explain how Thai LGBTQ+ adolescents experience or make sense of their struggles with depression, nor why rates are higher than in Western countries.

For example, a recent U.S. national survey found that 53% of LGBTQ_+_ young people reported symptoms of depression, 66% reported symptoms of anxiety, and 39% seriously considered suicide [4]. Similarly, an Australian national survey found that 59% had experienced a mental disorder for 12 months, 44% had high levels of psychological distress, and 48% had seriously considered suicide [4]. However, a national survey on the mental health of Thai children and youth with diverse sexual orientations and gender identities (SOGIESC) revealed that 71% of LGBTQ_+_ adolescents experienced depression, 78% anxiety, 58% suicidal thoughts, and 15% had attempted suicide [2]. While these findings highlight a serious public health concern, they also highlight that prevalence is impacted by location and cultural context.

Most existing literature emphasizes prevalence and risk factors rather than exploring the lived experiences of LGBTQ_+_ youth with depression within specific cultural contexts. Additionally, most studies are conducted in the Global North, limiting generalizability to countries in the Global South where legal rights for LGBTQ_+_ individuals are fewer, mental health stigma is greater, and cultural contexts differ [5,6,7,8]. Where research does emphasize the Global South, the focus is often on culture-bound syndromes, decolonial interventions, and service delivery [9]. In one study of programs in low- and middle-income countries aiming to reduce mental health stigmatization, only 20% considered culture in its application [7]. Thus, qualitative research from the Global South that focuses on cultural context will help fill the gaps and improve understanding of how to most effectively promote and serve LGBTQ_+_ youth health.

Several cultural and social factors shape the mental health experiences of LGBTQ_+_ adolescents. In Thailand, families differ from Western families in key dimensions, including prioritizing family over individual preferences, emphasizing parental authority, valuing family reputation, and maintaining privacy norms that influence the disclosure of personal experiences related to sexuality or gender [10,11]. These cultural characteristics affect how LGBTQ_+_ adolescents navigate traditional gender expectations and make decisions about disclosing their sexual orientation or gender identity.

Research from the Philippines also found that school-related challenges, including peer interactions and awareness among teachers or healthcare providers, also influence adolescents’ emotional well-being and access to support [12]. Depression among LGBTQ_+_ adolescents is recognized as a significant public health concern, particularly in rural schools and communities with limited mental health resources [2,10,13]. Although mental health services exist, they are often not tailored to the specific needs of LGBTQ_+_ adolescents, indicating the need for culturally responsive care and enhanced support system [14,15].

LGBTQ_+_ adolescents are particularly vulnerable to depression [1,2]. Minority Stress Theory highlights how stressors such as discrimination, expectations of rejection, and internalized stigma can affect mental health [16]. Although many studies have examined prevalence and risk factors, few have explored how Thai adolescents experience depression, navigate self-discovery, and access culturally relevant support [15]. In the Thai context, these experiences may be intensified by cultural values emphasizing conformity and family respect, suggesting that Thai LGBTQ_+_ adolescents face distinctive forms of minority stress compared with Western contexts [1,2]. Understanding these experiences is crucial, as culturally tailored interventions can foster resilience, improve mental health outcomes, and support positive identity development. This study addresses these gaps by investigating personal experiences, coping strategies, and support systems. The study results may potentially inform culturally grounded insights for mental health professionals, educators, and families.

To address the limitations of quantitative research in capturing the lived experiences of LGBTQ_+_ adolescents, the present study employs a phenomenological approach which focuses on understanding individuals’ lived experiences and the meanings they ascribe to them. Phenomenology is particularly suited for exploring complex processes such as depression, self-discovery, healing, and moving beyond symptom measurement to capture how adolescents embody and express emotional suffering and recovery. This approach also illuminates how cultural expectations, family relationships, and social stigma intersect with personal identity and coping strategies which can provide a nuanced understanding of the experiences of Thai LGBTQ_+_ adolescents [2,4]. By exploring lived experiences, researchers can uncover how adolescents find resilience, construct meaning from their identities, and identify support that facilitates or hinders healing, whether familial, peer-based, or professional. Such insights can inform culturally competent interventions and help reduce stigma in healthcare and educational settings, therefore aligning with broader calls in global mental health research for contextually grounded and culturally responsive studies [11,17].

This study seeks to understand how adolescents interpret and cope with depression in relation to their gender or sexual identity, how sociocultural and familial factors shape these experiences, and what types of support they perceive as essential for recovery. The study also considers how stigma, discrimination, and social pressures within Thailand’s collectivist culture, where family honor and conformity are highly valued, may influence adolescents’ mental health experiences, with insights from Minority Stress Theory [16] providing a supporting perspective. Applying phenomenology allows the research to move beyond identifying stressors to understanding how adolescents make meaning of their struggles and healing processes, highlighting the intersection of identity, culture, and mental health. By focusing on depression in this population, the study addresses a pressing issue recognized across families, schools, communities, and healthcare systems in Thailand, particularly in rural areas where support resources are limited. The findings aim to inform inclusive, culturally sensitive practices among families, healthcare providers, educators, and policymakers to better support the well-being of Thai LGBTQ_+_ adolescents.

## 2. Materials and Methods

This study employed Colaizzi’s phenomenological method, as outlined by Thomas and Pollio [18], to explore and interpret the lived experiences of Thai LGBTQ_+_ adolescents living with depression. This approach was chosen because it offers a systematic framework that balances descriptive clarity with interpretive depth and emphasizes grounding the analysis in participants’ narratives [19].

### 2.1. Sampling

Participants were purposively selected using a maximum variation sampling approach to capture diverse sexual and gender identities, including gay, lesbian, bisexual, transgender, queer, and non-binary adolescents. Inclusion criteria were ages 13–18, self-reported LGBTQ_+_ identity, mild-to-moderate depression (PHQ-A scores 5–14) [20], and the ability to articulate and reflect on their experiences, while participants with communication or language barriers were excluded. Mild-to-moderate depression was selected because adolescents in this range can safely participate in qualitative interviews, whereas those with severe symptoms may face higher emotional vulnerability and were therefore referred for specialist support. As interviews were conducted in Thai, fluency in Thai was required, and translations into English were completed and reviewed by bilingual researchers to ensure accuracy. This focus also aligns with national trends indicating that most Thai adolescents with depressive symptoms typically fall within the mild-to-moderate range, providing further justification for the inclusion criteria.

### 2.2. Procedures

#### 2.2.1. Recruitment and Assent

Recruitment was conducted in collaboration with school counselors in secondary schools under the Secondary Educational Service Area Office 11, Thailand. An initial online screening tool was sent to 617 students, and self-reported LGBTQ_+_ identity was confirmed during the screening. Of these, 20 adolescents met all inclusion criteria and consented to participate. No participants were excluded after enrollment. Preliminary conversations confirmed that all 20 participants could provide rich descriptions.

This sample was sufficient to capture diverse perspectives and achieve thematic saturation, defined as the point when no new themes emerged from successive interviews. A flow diagram of recruitment, screening, and enrollment was maintained to ensure transparency. Colaizzi’s method accommodates this range because of its emphasis on variation and verification through repeated comparison across participant narratives [19]. The high rate of refusal was carefully considered in the interpretation of results, as it underscores the deep stigma and vulnerability experienced by Thai LGBTQ_+_ adolescents. To safeguard ethical standards, recruitment and consent procedures were designed to protect participants’ privacy. Adolescents received study information directly from school counselors and contacted the researchers independently if they wished to participate. Parental consent and adolescent assent were obtained through a two-stage confidential process. Parents were informed that the study explored “adolescents’ emotional experiences,” without explicit mention of LGBTQ_+_ identity, to avoid unintentional outing of participants. Participants’ identities were protected with pseudonyms, private settings, and secure data storage, with safeguards against involuntary disclosure. Psychological support was available for participants experiencing distress, with protocols for severe reactions, including referral to mental health professionals or emergency services. In focus groups, participants could skip questions and confidentiality was emphasized. This approach aligns with international ethical guidelines such as the Belmont Report and the APA Ethics Code, which emphasize respecting participants’ autonomy, protecting vulnerable populations, and avoiding foreseeable harm, including risks related to involuntary disclosure of sexual or gender identity. Adolescents then provided written assent and were reminded of their right to withdraw from the study at any stage. Ethical approval was granted by the Human Research Ethics Committee of “MASKED FOR REVIEW,” and the study adhered to international standards for research involving minors and sexual minority populations.

#### 2.2.2. Data Collection

Data were collected from August to December 2023. Data collection involved semi-structured, one-on-one interviews and follow-up focus groups. Each participant took part in one in-depth interview lasting approximately 40–60 min and one focus group discussion lasting 60–75 min. Individual interviews elicited personal reflections and emotions, while focus groups explored shared meanings and collective coping strategies within the LGBTQ_+_ community. Each focus group consisted of six to seven participants, grouped primarily by age to facilitate comfort and openness. Interviews were conducted either face-to-face in private school counseling rooms or virtually through encrypted video calls, ensuring safety and accessibility. All sessions were audio-recorded with consent and transcribed verbatim in Thai. Transcripts were reviewed and verified by two members of the research team to ensure accuracy and completeness.

The semi-structured interview guide included five open-ended questions designed to encourage participants to share their narratives freely: (1) When did you first realize your LGBTQ_+_ identity, and how did it affect you?, (2) How do you think your experiences as an LGBTQ_+_ adolescent relate to your mood or emotional well-being?, (3) How do you cope with depression, and how did you get through that time?, (4) How can others, when aware of your identity or experiences, provide support that is helpful to you?, and (5) How can others better understand your experience and support you? The researchers built rapport before beginning each interview and used prompts to encourage participants to elaborate on their feelings and perspectives. Data collection and analysis occurred concurrently, allowing emerging insights to guide subsequent interviews and ensuring iterative exploration of emerging themes.

#### 2.2.3. Data Analysis

The data were analyzed using Colaizzi’s phenomenological method [19,21], which provided a structured yet flexible approach to understanding the lived experiences of LGBTQ_+_ adolescents. (1) All interviews were transcribed verbatim, and researchers repeatedly listened to recordings while reading transcripts to immerse themselves in each participant’s lived experience. (2) Significant statements related to identity, depression, and emotional experiences were extracted, capturing the essential meanings of participants’ experiences. (3) These statements were interpreted in context to formulate meanings, reflecting how adolescents made sense of and navigated emotional and identity-related challenges. (4) Related meanings were grouped and synthesized into overarching core structures that represent the essence of participants’ experiences, avoiding generic thematic labels. (5) A comprehensive description of the phenomenon was written, integrating these core structures to portray the complexity, resilience, and vulnerabilities of LGBTQ_+_ adolescents facing mental health challenges. (6) Reflexivity was applied throughout, with the research team critically examining how their own perspectives and experiences influenced interpretation, treating subjectivity as an integral part of meaning-making rather than bias. (7) Validation was achieved through member checking with participants and peer debriefing by external qualitative experts, confirming that the analysis authentically captured the essence of participants’ lived realities. This seven-step process maintained alignment with Colaizzi’s phenomenological philosophy, moving from individual narratives to the essential structures of experience while providing a rigorous and transparent approach to understanding identity formation, emotional struggle, and resilience among LGBTQ_+_ adolescents.

The research team consisted of five members with expertise in psychiatric and mental health nursing, family medicine, and community health from institutions in Thailand and the USA. The team received training and conducted pilot interviews to strengthen competence in working with adolescents on sensitive topics. As external researchers with no prior relationship or vested interest in the participants, their positions did not influence recruitment, interviews, or data interpretation. In addition, the emotional impact on the researchers was also acknowledged as a potential source of influence on data interpretation; however, this was addressed through systematic reflexive practices, including maintaining reflexive journals, engaging in regular peer debriefing, and using bracketing to separate personal reactions from analytic decisions. These strategies helped minimize bias and ensured that the findings remained grounded in participants’ narratives rather than researchers’ emotional responses.

Reflexivity was central to the study. Each researcher maintained a reflexive journal to document personal assumptions, emotional responses, and potential biases related to gender, sexuality, and mental health. Bracketing and regular team discussions were used to reflect on how their positionalities might shape interactions and data analysis, ensuring that reflexivity informed but did not bias the findings.

Trustworthiness of the study was established through credibility, transferability, dependability, and confirmability [22,23], ensuring that the findings genuinely represented the lived experiences of LGBTQ_+_ adolescents. Credibility was strengthened through prolonged engagement, member checking, and peer debriefing with two experienced qualitative researchers over three meetings spanning six weeks to review emerging themes and provide feedback on interpretations [18,23]. Member checking involved participants confirming conclusions drawn from their interviews. Transferability was supported by detailed contextual descriptions of participants and settings, allowing readers to assess applicability to other contexts. Dependability and confirmability were ensured through a meticulous audit trail of decisions, coding frameworks, and reflexive memos. Maximum variation sampling captured diverse perspectives, representing both common and unique experiences, which enhanced the depth and authenticity of the data. Analysis concluded when themes fully reflected participants’ experiences of living with depression, marking the culmination of a careful, transparent, and ethically rigorous qualitative inquiry.

## 3. Results

The study included 20 LGBTQ_+_ secondary school students aged 13–18 years (mean = 15.1, SD = 1.8), with most participants being 13–14 years old. Participants identified as lesbian (7 of 20), bisexual (8 of 20), gay (1 of 20), transgender (1 of 20), and queer (1 of 20). The majority of students lived with both parents (16 of 20), while a few lived with a single parent (4 of 20). Parental occupations were primarily general labor (10 of 20) and gardening (7 of 20), with one teacher and two participants’ parents in other occupations. Family relationships were mostly harmonious (14 of 20), with a few reporting distant (2 of 20), indifferent (1 of 20), or conflicted (1 of 20) interactions. These contextual details provide important background for understanding the participants’ experiences of living with depression.

This study identified three key themes reflecting the experiences of LGBTQ_+_ adolescents with depression: (1) struggles with self-discovery, acceptance, and their relationship to depression; (2) living with depression and moving forward; and (3) the need for acceptance and understanding. Each theme encompasses several sub-themes that offer nuanced insights into the emotional, psychological, and social aspects of their experiences. These findings underscore the complex interaction between identity development, emotional health, and social support in shaping mental health outcomes among LGBTQ_+_ youth.

### 3.1. Struggles with Self-Discovery, Acceptance, and Their Relationship to Depression

The journey of self-discovery and acceptance is a profound and often painful process for LGBTQ_+_ adolescents, especially when societal norms and fear of rejection create persistent internal conflicts. Hiding one’s true identity to avoid judgment not only affects daily interactions but can also deepen depressive feelings. This theme is explored through the following subthemes: Section 3.1.1. Struggle for self-acceptance and the fear of rejection and Section 3.1.2. Hidden struggle of gender identity and depression.

#### 3.1.1. Struggle for Self-Acceptance and the Fear of Rejection

This subtheme examines the tension between personal identity and societal expectations, highlighting the internal conflicts and emotional toll that arise when LGBTQ_+_ adolescents attempt to reconcile who they are with how they are perceived.

##### Internal Conflict Between Self-Acceptance and Societal Pressure

LGBTQ_+_ adolescents often experience a deep internal struggle as they attempt to align their authentic identity with societal expectations, leading to confusion, guilt, and emotional distress. The pressure to conform can feel inescapable, reinforcing feelings of difference and inadequacy.

“*…Since elementary school, I realized I’m neither the girl nor the boy others expect. Being around girls feels right, but my male friends bully me. I tried to act like a girl, but it never felt right. The more I understood, the harder it became to accept myself. Society tells me who I should be, but deep down, I can’t fit that. The struggle to accept myself feels impossible when everything around me says I’m different…*”

(P5)

“*…I’ve always known I was different, but society told me I had to be something I’m not. I was forced to act a certain way, but inside, I felt like I was lying. I want to be myself, but I’m scared I’ll never be accepted if I do. Every day feels like a battle between who I am and who everyone expects me to be…*”

(P16)

##### Fear of Rejection from Family and Friends After Self-Realization

The fear of rejection from loved ones often forces LGBTQ_+_ adolescents to conceal their identity, creating a persistent emotional conflict. This concealment leads to isolation and tension in relationships as adolescents weigh the desire for authenticity against the risk of being unloved or judged.

“*…I’ve always feared that if my family knew the real me, they might not accept me and feel disappointed. Every time I try to bring it up, they avoid the topic, and I fear disappointing them. I feel like I have to hide who I am just to keep things peaceful, and the fear of rejection never really goes away…*”

(P7)

“*…I’m terrified of my friends finding out the real me. They already joke about what they don’t understand. The thought of being rejected by those I trust most keeps me hiding who I am. I’m always pretending, just to fit in…*”

(P2)

#### 3.1.2. Hidden Struggle of Gender Identity and Depression

This subtheme highlights how struggles with gender identity are deeply intertwined with mental health, showing that societal rejection, bullying, and internalized stigma can intensify depressive symptoms.

##### Depression as a Response to Rejection, Bullying, and Social Stigmatization

Depression among LGBTQ_+_ adolescents often emerges as a response to external invalidation and social stigmatization. Experiences of teasing, exclusion, and misunderstanding not only impact daily life but also foster a persistent sense of unworthiness and emotional isolation.

“*…Every time someone mocks me or looks at me like I’m strange, it feels like a weight on my chest. I hide who I am, afraid of being judged. The teasing and lack of understanding wear me down, making me wonder if I’ll ever belong or if this sadness will always be a part of me…*”

(P5)

“*…The more I try to be myself, the more people push me away. I feel invisible, and the bullying only makes it worse. It’s hard to keep going when all I hear is that I’m wrong or not good enough… It feels like I’m living in a world that isn’t mine.*”

(P2)

##### Silent Struggles of Depression in LGBTQ_+_ Adolescents

LGBTQ_+_ adolescents often conceal their depressive experiences to avoid judgment, resulting in intensified feelings of loneliness and emotional burden. The pressure to hide one’s authentic self compounds sadness, creating a silent, persistent struggle.

“*…I smile every day to hide the sadness inside. No one knows what I’m going through because I’m afraid of being judged. I feel trapped by the expectations of others but can’t bring myself to talk about it. Every time the sadness hits, I keep it to myself, letting it grow deeper with no way out…*”

(P11)

“*…My depression is no different from anyone else’s, but when it comes to my identity, it makes it worse. The rejection and judgment about my sexuality makes everything feel heavier, like my sadness is amplified by not being accepted for who I really am…*”

(P10)

### 3.2. Living with Depression and Moving Forward

Participants described living with depression as a fluctuating experience, marked by shifts between emotional heaviness and moments of clarity. Rather than progressing in a linear way, they continually negotiated between internal distress and the strategies that helped them stay grounded. These dynamics are reflected in two subthemes: Section 3.2.1. Coping strategies for managing depression and Section 3.2.2. Support and connection in overcoming depression.

#### 3.2.1. Coping Strategies for Managing Depression

Participants shared diverse ways of coping, often shifting between self-reliance and reaching out for support. These strategies were not described as solutions, but as ways of “holding themselves together” during periods of emotional difficulty.

##### Using Mindfulness to Heal and Overcome Depression

Some participants used practices that helped them return to themselves when emotions felt overwhelming. Mindfulness, reflection, or slowing down were described less as techniques and more as moments of reclaiming a sense of inner steadiness amid distress.

“*…When I’m really down, I focus on staying with myself, just breathing and letting the sadness pass*. *I try not to care about what others think or say*. *It’s hard, but I remind myself that I can get through this moment, no matter how deep it feels…*”

(P13)

“*…I first check in with myself—understanding my emotions and taking care of my thoughts*. *I reflect and practice self-care before reaching out to friends for support*. *I look up ways to manage depression on my own, and only talk to people I trust when I need it*. *I avoid opening up to adults or teachers who might judge me…*”

(P15)

##### Embracing Self-Acceptance and Moving Beyond Societal Pressures

Participants described self-acceptance as emerging gradually, often through tension between who they felt they were and what society expected of them. Rather than a final state, self-acceptance appeared as an ongoing movement strengthened at times, shaken at other times, and shaped by daily encounters with stigma and support.

“*…I’ve learned to accept myself for who I am, even when the world tells me I should be different. It’s not easy, but I keep reminding myself that my worth isn’t defined by anyone’s expectations. I focus on doing what feels right for me, and let go of the pressure to fit into their mold…*”

(P3)

“*…The pressure to fit society’s expectations used to hold me back, especially with bullying and discrimination. Now, I block out the negativity and focus on what matters. I surround myself with those who accept me and remind myself I don’t need others’ approval. It’s tough, but I keep moving forward by staying true to who I am…*”

(P1)

#### 3.2.2. Support and Connection in Overcoming Depression

Support from others—whether consistent or occasional—plays a significant role in how adolescents make sense of their depression. Such support did not eliminate struggles but provided moments of validation that softened feelings of isolation and strengthened their ability to continue.

##### Experiencing Affirmation Through Family Acceptance and Professional Support

Participants described family acceptance or professional understanding as deeply impactful. These encounters provided a rare sense of being seen without judgment, allowing adolescents to momentarily step out of the emotional weight of rejection.

“*…I used to worry so much about my parents accepting me. One day, a neighbor made a hurtful comment, and my family just said, ‘So what if you’re different? It doesn’t matter.’ In that moment, I felt relieved. If my family accepts me, nothing else matters.*”

(P3)

“*…Seeking professional help was a turning point. The expert didn’t need to be LGBTQ_+_, just some-one who listens, understands, and doesn’t judge. It made me realize my feelings are valid and that I’m just as normal as anyone else, helping me move forward with more clarity in my identity…*”

(P6)

##### Finding Strength in Supportive Friendships and Shared Experiences

Friends and peers provided a sense of belonging that countered isolation. For many, sharing experiences with others who faced similar struggles created a space where vulnerability felt safer and depression felt less like a solitary burden.

“*…Having friends who truly understand and accept me has been a lifeline. They make me feel valued, and it’s comforting to know I have people who support me, offering a space where I can just be myself without fear of judgment…*”

(P9)

“*…Being around friends who are going through the same struggles makes all the difference. We talk openly about our feelings, and it feels like we’re in this together. It’s easier to move through the tough times when you know someone else truly gets it…*”

(P8)

### 3.3. The Need for Acceptance and Understanding

For LGBTQ_+_ adolescents, acceptance and understanding from family, school, and society emerged not simply as external support but as core conditions shaping how they made sense of themselves, managed internalized stigma, and navigated emotional vulnerability. Their narratives reflected a tension between longing for recognition and fear that visibility might invite harm, revealing the complexity underlying requests for acceptance. Two subthemes illustrate these dynamics: Section 3.3.1. Familial acceptance as a key to emotional healing and Section 3.3.2. Creating safe spaces in schools and society.

#### 3.3.1. Familial Acceptance as a Key to Emotional Healing

Familial acceptance plays a central role, not just as a form of support, but as a relational space in which adolescents negotiate feelings of worth, identity legitimacy, and internal conflict. Acceptance was often described as a powerful emotional relief, yet it also coexisted with a lingering fear of losing that acceptance.

##### Parental Support Impacts Mental Health and Emotional Well-Being

Participants described parental acceptance as a moment of emotional release that allowed them to reorient their sense of self, reducing internalized stigma and enabling greater openness. Yet even positive experiences contained traces of uncertainty about whether this acceptance would remain stable.

“*…When my parents finally accepted me, it felt like a weight lifted off my shoulders. It was like being freed from a small, suffocating space—like stepping out of a locked closet. Their acceptance gave me the freedom to truly be myself…*”

(P18)

“*…Nothing else matters as much as family when it comes to healing. If the people I love the most truly understand and accept me for who I am, it feels like everything else falls into place. Their love and support are the foundation that helps me move through the pain…*”

(P4)

##### Difficulties Faced When Parents Are Not Accepting or Understanding

Experiences of non-acceptance were not only painful but also shaped adolescents’ internal landscapes, creating cycles of guilt, self-blame, and internalized stigma. Participants often described ambivalence-wanting to disclose yet fearing emotional consequences, which deepened their distress.

“*…The fact that my parents can’t accept me makes me feel hurt. Every time I try to open up, I see disappointment and avoidance in their eyes. It hurts more than any words could, and it makes me feel guilty for disappointing them. It’s like being rejected by the people who are supposed to love me unconditionally…*”

(P5)

“*…When my parents don’t understand, it feels like I’m invisible, like I’m living a life they can’t see or acknowledge. Their silence or judgment makes me question my worth, and it’s hard to move forward when the people I need the most aren’t there to support me…*”

(P17)

#### 3.3.2. Creating Safe Spaces in Schools and Society

Safe spaces were described as contexts in which adolescents could temporarily set aside the vigilance and self-monitoring demanded in daily life, allowing them to explore their identities with less fear. Yet even within supportive environments, some students expressed uncertainty about how fully they could be themselves, reflecting the ongoing negotiation between safety and risk.

##### Role of Schools in Creating Inclusive and Supportive Environments

Schools that offered structured support (e.g., clubs, affirming teachers) provided a reprieve from stigma and allowed adolescents to experiment with self-expression. However, this sense of safety was sometimes fragile, shaped by concerns about peers’ judgments or inconsistent teacher support.

“*…A space like the Rainbow Club at school makes me feel less alone, like I have friends who are going through the same experiences. It shows that the school recognizes us as a normal part of society, just like any other group…*”

(P12)

“*…Schools can create safe spaces where LGBTQ_+_ students feel secure. Teachers should act as intermediaries, helping parents and society understand that being LGBTQ_+_ is normal, and science has proven that the world is not just made up of male and female. Proper support and communication can make all the difference…*”

(P14)

##### Importance of Public Awareness Campaigns in Promoting Understanding

Participants perceived society’s limited understanding as a persistent source of distress, contributing to conflicting feelings of pride and fear, and reinforcing internalized stigma. Public awareness efforts were seen as meaningful primarily because they could shift everyday interactions, reducing the sense of being watched, judged, or misunderstood.

“*…Although the world is becoming more open, LGBTQ_+_ still feels unclear, hidden in a fog. We see ourselves as normal, but society labels us as fragile. Gender and sexual orientation exist on a spectrum, so why limit it to just two? The lack of understanding from society hurts us, but LGBTQ_+_ is normal, and it needs to be recognized….*”

(P19)

“*…People need to stop viewing LGBTQ_+_ as strange. It’s simply part of who we are. Public awareness campaigns are key in breaking down barriers, reducing stigma, and showing there’s nothing to fear. Understanding and ending society’s prejudice allows us to move forward with dignity, without harming anyone.*”

(P20)

## 4. Discussion

The lived experiences of Thai LGBTQ_+_ adolescents revealed a complex web of struggles and triumphs on their journey toward self-discovery, healing from depression, and seeking support. These experiences are deeply interconnected with issues of familial acceptance, societal pressures, and the need for peer and public support. The study identified three core themes: Section 3.1. “Struggles with Self-Discovery, Acceptance, and Their Relationship to Depression, “ Section 3.2. “Living with Depression and Moving Forward,” and Section 3.3. “The Need for Acceptance and Understanding,” which all offer insight into how these adolescents navigate their emotional challenges.

Meta-analysis of previous qualitative studies found similar themes involving navigating one’s identity, the need for belonging locally and within society, as well as internal coping experiences [8,24]. Meta-analysis of the worldwide prevalence of major depressive disorder in LGBTQ_+_ individuals has also been averaged at 32.2% globally [25]. This study extends previous research by highlighting how cultural values and evolving gender and sexuality identities shape experiences of depression among Thai LGBTQ_+_ adolescents, particularly in rural areas—an aspect less explored in prior literature [26,27,28]. While studies in other countries have examined childhood adversities and mental health in LGBTQ_+_ youth, this study provides context-specific insights on Thai cultural constructs such as “bunkhun” and conditional parental acceptance, showing how these uniquely affect emotional well-being [15,29,30].

The first theme, “Struggles with Self-Discovery, Acceptance, and Their Relationship to Depression,” highlights the profound internal conflict LGBTQ_+_ adolescents face in reconciling their authentic selves with societal expectations. Support environments not only serve as a protective factor; they also help facilitate genuine exploration of one’s identity [24]. This suggests that the absence of supportive environments may also slow or stunt self-acceptance. In Thailand, this conflict is shaped not only by social norms but also by deeply rooted cultural values surrounding family, particularly the role of parents as primary influencers of emotional and psychological development [10,11]. Globally, family acceptance is an established protective factor for negative health outcomes, such as depression, suicidality, and drug abuse [5,8,31]. However, while Thailand projects an image of sexual diversity tolerance, acceptance often remains superficial and conditional [2,11], creating emotional confusion and reinforcing shame.

Expectations to conform to heteronormative roles have been shown to intensify mental health struggles [28]. This pressure is further complicated in Thailand by cultural concepts like “bunkhun,” where disappointing parents is internalized as moral failure, adding to psychological distress [10,29]. Studies consistently link such familial tension with depression, anxiety, and self-harm in LGBTQ_+_ youth [31,32,33]. The significance of familial rejection is also apparent by the disproportionate number of homeless youth who identify as LGBTQ_+_ [8]. Although emerging family education programs and community-based dialogues attempt to bridge this gap, these remain limited and require broader structural support from educational and policy systems [29]. This study demonstrates how these culturally specific pressures intersect with universal challenges observed in global LGBTQ_+_ populations.

In the second theme, “Living with Depression and Moving Forward,” participants described the emotional challenges associated with depression, including feelings of isolation and stress related to societal expectations about gender and identity. In the Thai context, adolescents highlighted the influence of local cultural norms and community perceptions on their emotional experiences, including the role of family expectations and school environments in shaping how adolescents access support and develop coping strategies. Recognizing and accepting their authentic selves emerged as important steps toward emotional well-being.

LGBTQ_+_ individuals tend to employ self-management or coping strategies more often compared to heterosexual peers, including seeking support from family or friends, positive thinking, and setting achievable goals [34]. Coping strategies reported by this study’s participants included mindfulness, emotional expression in supportive settings, and emotional self-regulation, which helped them understand and manage their feelings while building resilience [14,26]. Support from families, friends, and mental health professionals, such as counselors or psychologists, contributed to a sense of connection and emotional support [28,35].

In countries where cultural or religious norms strongly influence family and community attitudes toward LGBTQ_+_ adolescents, strategies that emphasize safe and confidential spaces for support, culturally sensitive counseling, and peer networks can help youth build resilience and maintain well-being. These safe and confidential spaces are particularly important as the degree to which an adolescent is “out” influences their perceived ability to get support [36]. These approaches can be adapted to align with local values while promoting acceptance of diverse identities and supporting mental health [1].

For example, supportive online communities have been found to be especially beneficial for those who are either geographically isolated or who are either not “out” or who are not supported by their families to attend support groups [8]. However, for those in collectivist contexts, anonymity might be a crucial element for online groups. In one study, Hispanic LGBTQ_+_ individuals preferred anonymity when seeking social support on social media, whereas their Black and non-Hispanic White participants did not. The cultural value of familialism—the belief that family must remain physically and emotionally close whenever possible—may be a driving factor in this preference [37]. Anonymity is also seen more broadly through compartmentalizing one’s social media presence by creating alternate identities to safeguard against accidental disclosure. Combining these strategies with culturally relevant approaches can guide interventions and support systems tailored to Thai LGBTQ_+_ youth as well as provide guidance for contexts with different cultural or religious frameworks.

The third theme, “The Need for Acceptance and Understanding,” underscores the critical importance of familial, peer, and societal acceptance in shaping mental health outcomes for LGBTQ_+_ adolescents. Participants consistently reported that emotional well-being improved when parents, peers, and teachers expressed understanding and support [15,26]. Familial acceptance allowed adolescents to feel secure and valued, while supportive peers helped reduce feelings of isolation [8]. Schools providing inclusive environments, such as LGBTQ_+_ clubs or teacher advocacy, were also essential in fostering resilience, promoting a sense of belonging and protecting against depression [8,24,31,36].

The absence of inclusive environments in schools, healthcare, or community places is a double-edged sword. On the one hand, absence has been shown to stimulate personal agency by seeking out sexual health information online and developing effective self-management strategies [36,38]. On the other hand, silence or “benign neglect” regarding LGBTQ_+_ topics was found to reinforce shame and signal that expressing identities was unsafe [39]. However, when inclusive spaces are in place, such as public awareness campaigns, educational programs, and family education initiatives, they have emerged as important strategies to reduce stigma and enhance understanding of LGBTQ_+_ experiences [8].

This has been seen most clearly in the decline of suicide attempts, bullying, and negative mental health symptoms after states have instituted same-sex marriage laws, antibullying policies, and inclusive school safety strategies [38,40,41,42]. Inversely, after schools and public health supports were closed during COVID-19, the rates of mental health symptoms of LGBTQ_+_ youth doubled that of their heterosexual peers [6]. However, the tension between longing for recognition in safe spaces and fear that visibility might invite harm is a tenuous one.

Within healthcare settings and mental health services in particular, there is evidence that LGBTQ_+_ individuals are less likely to access services if they suspect the possibility of discrimination [36]. However, there are clear barriers and facilitators to healthcare access for the LGBTQ_+_ population that can help inform what makes a safe space in other settings as well. Barriers to healthcare access include: perceived lack of knowledge, risk of “outing,” the need for inclusive administrative processes, no visibility or the lack of visibility of LGBTQ_+_ affirming health services, phobic behaviors among clinicians, and assumptions of heteronormative identity. Conversely, facilitators to healthcare access include: adequate visibility of LGBTQ_+_ friendly practices (such as signage or flags), openly advertising competence, acceptance of described identities, proactive and proper use of pronouns, appropriate exploration of the relationship between identity and mental health, and the presence of LGBTQ_+_ staff [43,44,45]. Thus, a very practical application of these findings is to implement both policies and competency training for providers, teachers, and healthcare professionals.

For schools, providing LGBTQ_+_ clubs, inclusive sex education and inclusive history are tangible steps that reduce the fear of harm from visibility and increase a sense of inclusion [42]. Providing clear circumstances that enable action, descriptions of offensive or prohibited behaviors, and setting timelines for implementation or education have been shown to clearly decrease the odds of negative mental health outcomes [41] and also empower providers to foster inclusive environments.

This study adds novel insight by showing how cultural factors, such as family expectations, community norms, and differences between rural and urban settings in Thailand, shape the effectiveness of supportive interventions, providing context-specific evidence that complements existing international literature on the mental health of LGBTQ_+_ youth. By highlighting these culturally influenced dynamics, the findings emphasize the importance of contextually tailored interventions that foster emotional well-being and resilience among LGBTQ_+_ adolescents in Thailand [33,45].

Overall, this study provides new evidence on how cultural understanding, evolving gender and sexuality identities, and rural-urban contexts shape Thai LGBTQ_+_ adolescents’ experiences of depression [1,11]. Unlike prior research, which has largely focused on the Global North or urban areas, this study highlights how cultural expectations and limited resources intersect with universal mental health challenges [26,28]. These findings inform culturally sensitive interventions, inclusive mental health resources, and future research aimed at supporting LGBTQ_+_ adolescents in Thailand and similar cultural contexts, while recognizing their agency in promoting their own well-being [3,11].

## 5. Limitations

This study has several limitations. First, the participants were self-selected, which may represent a subgroup of Thai LGBTQ_+_ adolescents with greater support; therefore, findings should be interpreted with consideration of transferability rather than generalizability. Second, interviews were conducted in Thai and later translated into English for analysis; therefore, some cultural nuances may have been lost or altered. Third, data were collected at a single point in time, and changes in social attitudes or personal circumstances are not captured. Fourth, Minority Stress Theory has been criticized for not fully capturing all forms of stress that are encountered on a daily basis, including micro-aggressions and the concept of emotional labor—the daily practice of regulating and aligning one’s emotions with societal expectations regardless of one’s true feelings [24].

Despite these considerations, the study provides rich insights into the lived experiences of Thai LGBTQ_+_ adolescents, highlighting their identity development, emotional struggles, and coping processes. Future research with larger and more diverse samples, as well as longitudinal or mixed-method designs, is recommended to enhance the understanding and applicability of findings.

## 6. Conclusions

This study revealed three interconnected processes shaping Thai LGBTQ_+_ adolescents’ experiences of depression: struggles with self-discovery, acceptance, and their relationship to depression, living with depression and moving forward, and the need for acceptance and understanding. While some experiences mirror global LGBTQ_+_ mental-health patterns, this study demonstrates that Thai cultural norms, such as obligations to family reputation, expectations of conformity, and conditional acceptance, intensify minority stress in distinctive ways.

Support strategies must be culturally tailored to provide: confidential counseling for youth who cannot disclose at home, LGBTQ_+_-inclusive school programming, family education to reduce conditional acceptance, and community-based efforts that align with local cultural and religious frameworks. Effective interventions must include collaboration with supportive community and/or faith leaders, culturally sensitive psychoeducation, access to anonymous peer networks (including online spaces), and clear institutional policies that reduce stigma. Mental-health professionals, educators, and nurses can apply these findings by creating visibly affirming environments, incorporating LGBTQ_+_ cultural competence into care, and advocating for structural changes in schools and communities.

By implementing these strategies, support systems can help reduce stigma, strengthen resilience, and promote emotional safety for LGBTQ_+_ adolescents. This study advances current knowledge by demonstrating how cultural values, rural–urban differences, and conditional acceptance uniquely shape depression experiences in Thailand, offering evidence for interventions tailored to collectivist settings.

## Data Availability

The datasets presented in this article are not publicly accessible due to privacy constraints. To request access to the datasets, please contact Montha Saithanwanitkul and Saifon Aekwarangkoon.
